# Supplementary material for: Monitoring SARS-CoV-2 variants alterations in Nice neighborhoods by wastewater nanopore sequencing
Source: Lancet Reg Health Eur. 2021 Aug 17;10:100202. doi: 10.1016/j.lanepe.2021.100202 (PMC8372489; doi:10.1016/j.lanepe.2021.100202)
Supplement: Supplementary file 1 [file mmc1.pdf]

Figure 1

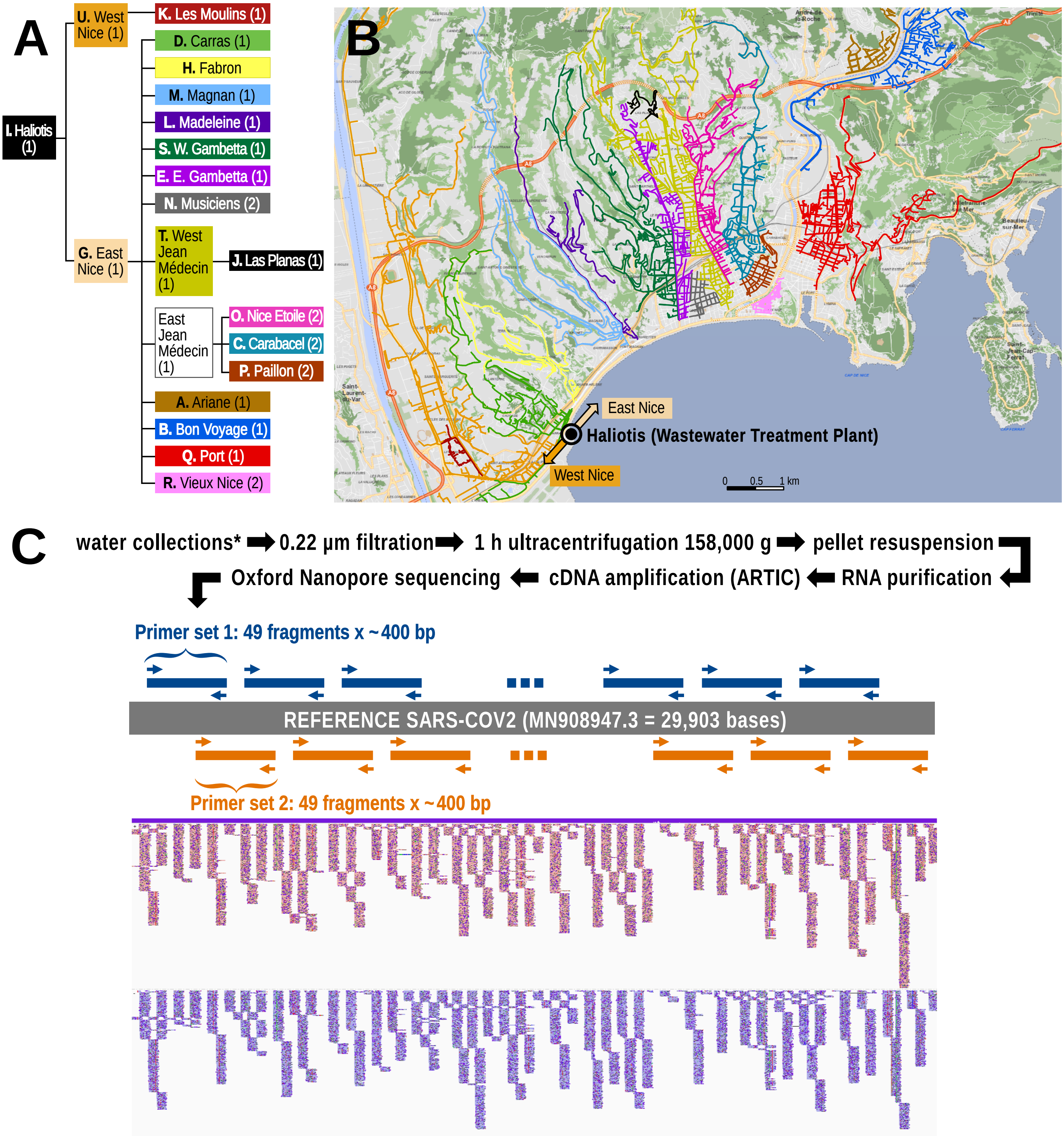

Figure 1: Experimental design of the study. (A) Hierarchical organization of the sampling points. **(B)** Map of the Nice area, with the indications of the different catchment areas. Haliotis is the name of the central wastewater treatment plant (WWTP). **(C)** flow-chart of the project, with the different steps of analysis. The ARTIC sequencing protocol is based on a polymerase chain amplification of 2 sets of non-overlapping amplicons that cover the full sequence of the virus.

Figure 2

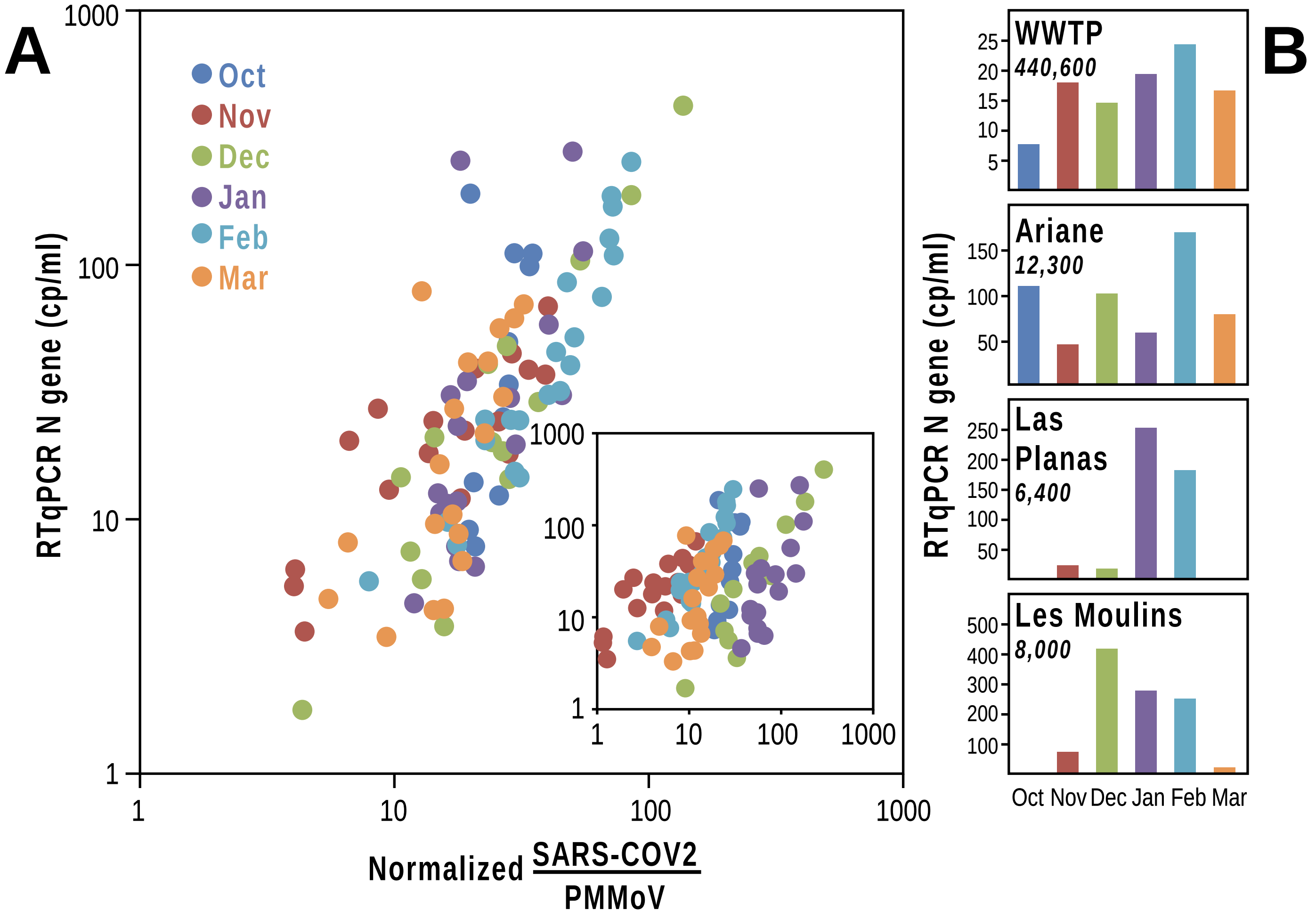

Figure 2: Comparison between RTqPCR and sequencing quantifications. (A) Relationship between normalized SARS-CoV-2 / PMMoV ratio and the average RTqPCR signal for the N gene. For each sampling date, a correcting ratio was defined by the weighted average signal between the different sampling points in the wastewater treatment plant (detailed in Supplementary Table 2). The inset shows the relationship between the SARS-CoV-2 / PMMoV ratio and the average RTqPCR signal for N gene before normalization. **(B)** N gene RT-qPCR Cq values were used to assess the concentrations of virus in the WWTP (Haliotis, Nice wastewater treatment plant) and the different neighborhoods. Values are provided in copies of genomes per ml of wastewater (cp/ml). Results are shown for 4 areas. Full results are provided in Supplementary Table 2. The population in each neighborhood is indicated under each name in italic.

Figure 3

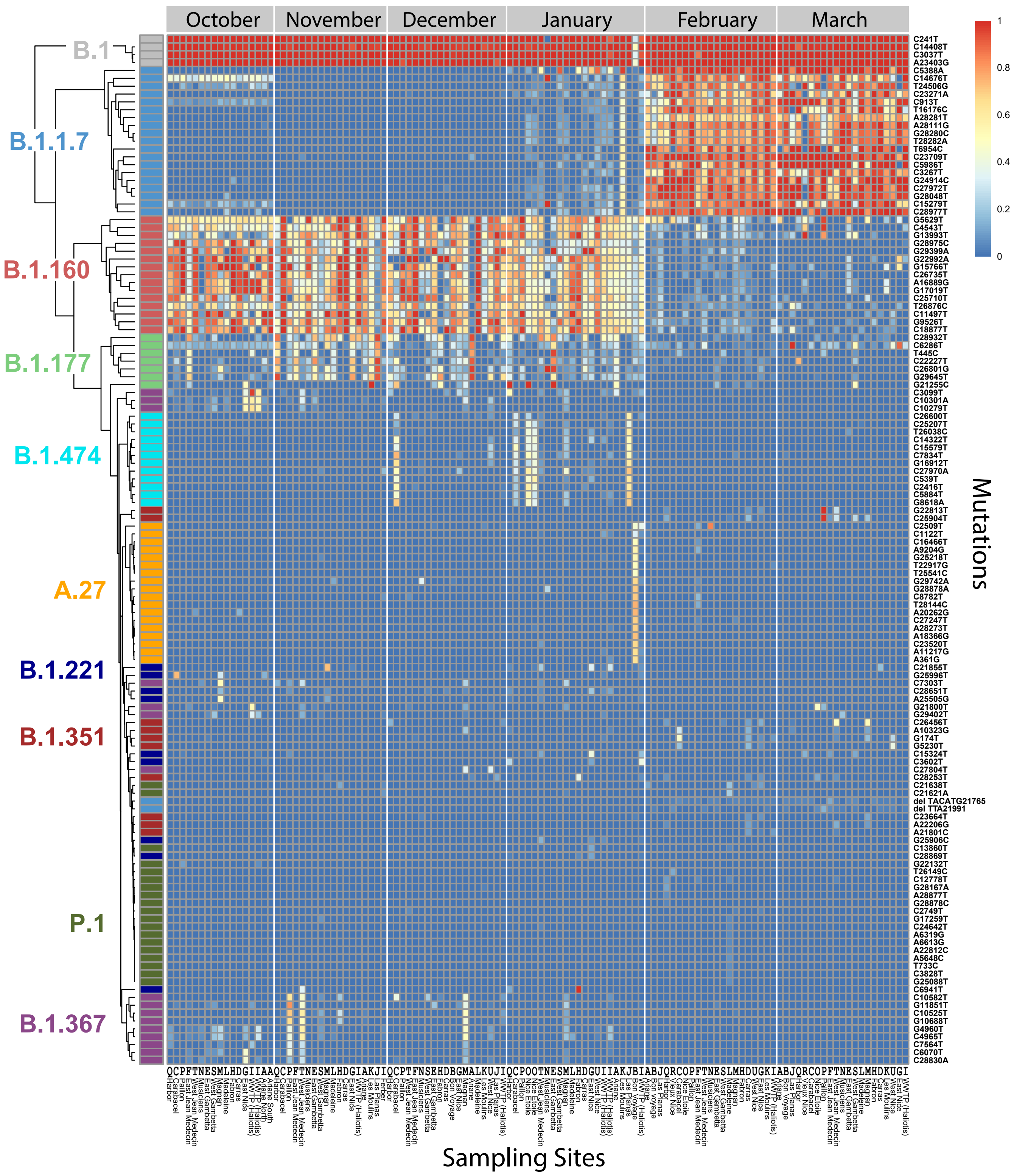

Figure 3. Mutation frequencies for the polymorphisms that define the ten most abundant lineages. Heatmap representation for ten lineages, grouped by dates of collection. For each lineage, the mutations characteristic for the lineage are shown (total: 179 mutations, rows) for each month and sampling site (columns). The color of the boxes indicates the fraction of the reads with the given mutation (blue 0%, red 100%). Reference of the different sampling sites (columns): A, Ariane; B, Bon Voyage; C, Carabacel; D, Carras, E, East Gambetta; F, East Jean Médecin; G, East Nice; H, Fabron; I, WWTP Haliotis; J, Las Planas; K, Les Moulins; L, La Madeleine; M, Magnan; N, Musiciens; O, Nice Etoile; P, Paillon; Q, Harbour; R, Vieux Nice; S, West Gambetta; T, West Jean Médecin; U, West Nice.The same letter code is used in Figures 1 and 4.

Figure 4

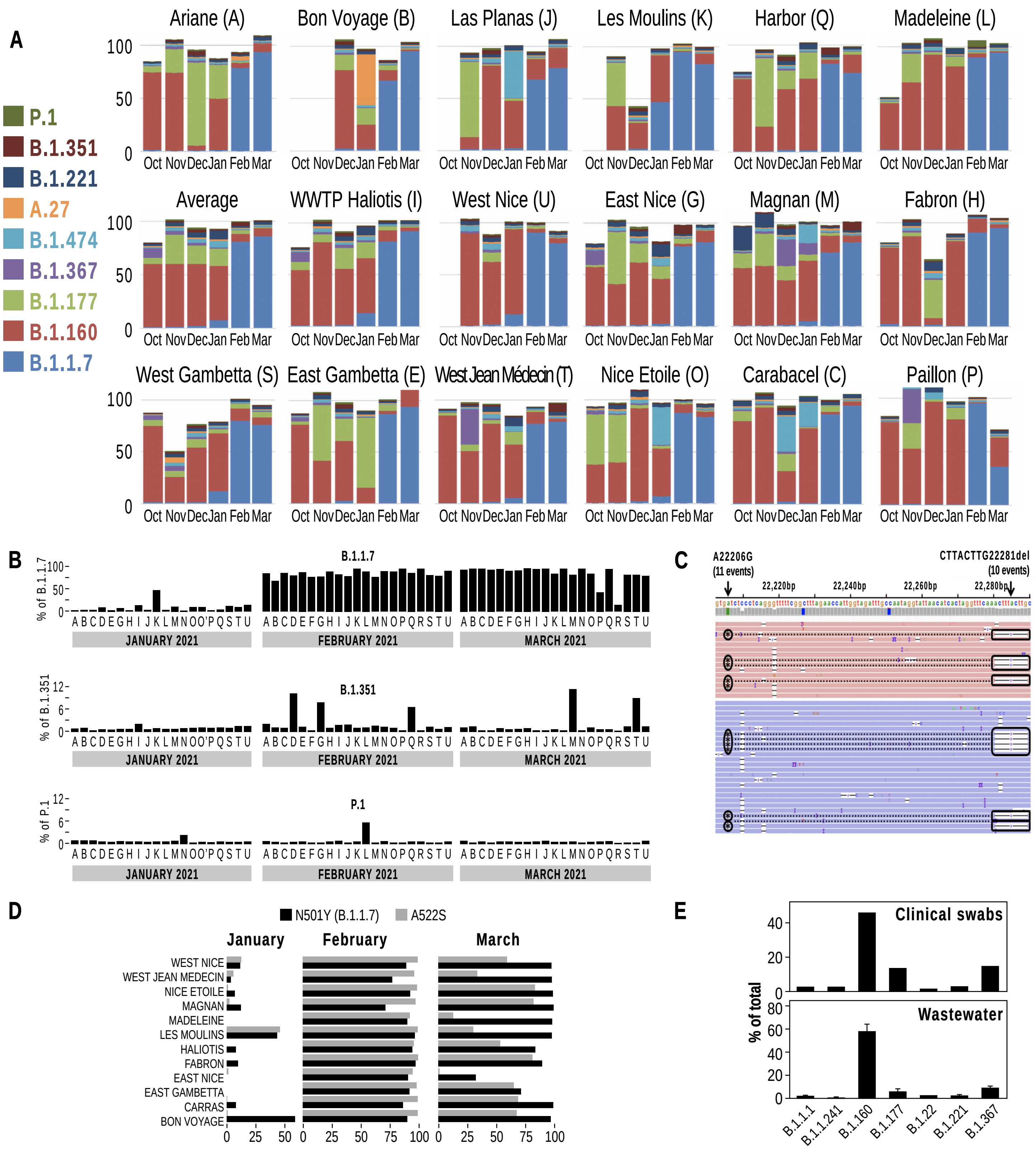

Figure 4. Characterization of SARS-CoV-2 lineages in the 113 samples. (A) Barplots illustrating the relative abundance of 9 lineages in the Haliotis WWTP and 17 neighborhoods. **(B)** Barplot showing the fraction of the B.1.1.7, B.1.351 and P.1 lineages in the different Nice neighborhoods. The correspondence between the letter code and the different neighborhoods is provided in Figure 1 and in the legend of Figure 3. Supplementary Figure 3 shows additional barplots for B.1.525 and A.23.1. **(C)** Association of two B.1.351 mutations in the same read. Shown for a sample from "Magnan" collected in March 2021. **(D)** Identification of a B.1.1.7 variant, characterized by the presence of an additional A522S (G23126T) mutation in the Spike protein. **(E)** Comparison of the frequencies of different lineages in clinical and wastewater samples both from week 43 (October 2020). Error bars for wastewater data represent SD of the frequencies of the mutations characterizing the variant. See also Supplementary Table 4.
