# Supplementary material for: Monitoring SARS-CoV-2 variants alterations in Nice neighborhoods by wastewater nanopore sequencing
Source: Lancet Reg Health Eur. 2021 Aug 17;10:100202. doi: 10.1016/j.lanepe.2021.100202 (PMC8372489; doi:10.1016/j.lanepe.2021.100202)
Supplement: Supplementary file 3 [file mmc3.docx]

**French Abstract:**

**Contexte :** La surveillance des eaux usées apparait comme un outil épidémiologique prometteur pour définir la prévalence et suivre l'évolution des épidémies de SRAS-CoV-2 sur un territoire. Jusqu’à présent, la plupart des projets de surveillance du SRAS-CoV-2 dans les eaux usées reposaient sur une mesure des titres de virus par PCR quantitative et ne fournissaient pas une vision exhaustive de toutes les mutations du SRAS-CoV-2 pouvant circuler dans la population.

**Méthodes :** Nous avons mis en place un système de surveillance par séquençage de l’ARN viral sur séquenceur Oxford Nanopore à l’échelle de toute la ville de Nice (France, 550 000 habitants). Entre octobre 2020 et mars 2021, nous avons analysé chaque mois les différents variants du SRAS-CoV-2 à partir de 113 échantillonnages d'eaux usées collectés au niveau de la station centrale d'épuration et de 20 quartiers différents de la ville.

**Résultats :** Nous avons initialement détecté les lignées prédominant en Europe à la fin de 2020 (B.1.160, B.1.177, B.1.367, B.1.474 et B.1.221). En janvier, l’émergence d'un variant de la lignée B.1.1.7 s'est produite dans un des quartiers de la ville, caractérisé par une mutation A522S sur la spicule. Ce variant s'est rapidement répandu dans toute la ville où il est devenu dominant à partir du mois de février. D'autres variants préoccupants (B.1.351, P.1) ont également été détectés dans certains quartiers, mais toujours à une faible fréquence. La comparaison avec les échantillons cliniques individuels collectés au cours de la même semaine a montré que le séquençage des eaux usées identifiait correctement les mêmes lignées que celles trouvées chez des patients COVID-19.

**Interprétation :** Le séquençage des eaux usées a permis de documenter de façon très précise la diversité des séquences du SRAS-CoV-2 présentes dans les différents quartiers de la ville de Nice. Nos résultats illustrent comment le séquençage des échantillons d'eaux usées peut être utilisé comme outil « micro-épidémiologique » pour suivre la dissémination d’agents pathogènes lors d’épisodes pandémiques et/ou épidémiques, présents ou futurs.
